# Biological Activity of CXCL8 Forms Generated by Alternative Cleavage of the Signal Peptide or by Aminopeptidase-Mediated Truncation

**DOI:** 10.1371/journal.pone.0023913

**Published:** 2011-08-31

**Authors:** Anneleen Mortier, Nele Berghmans, Isabelle Ronsse, Karolien Grauwen, Steve Stegen, Jo Van Damme, Paul Proost

**Affiliations:** The Laboratory of Molecular Immunology, Department of Microbiology & Immunology, Rega Institute, Katholieke Universiteit Leuven (K. U. Leuven), Leuven, Belgium; University of Brescia, Italy

## Abstract

**Background:**

Posttranslational modification of chemokines is one of the mechanisms that regulate leukocyte migration during inflammation. Multiple natural NH_2_-terminally truncated forms of the major human neutrophil attractant interleukin-8 or CXCL8 have been identified. Although differential activity was reported for some CXCL8 forms, no biological data are available for others.

**Methodology/Principal Findings:**

Aminopeptidase-cleaved CXCL8(2-77) and CXCL8(3-77), the product of alternative cleavage of the signal peptide CXCL8(-2-77) and the previously studied forms containing 77 and 72 amino acids, CXCL8(1-77) and CXCL8(6-77), were prepared by solid-phase peptide synthesis, purified and folded into active proteins. No differences in binding and calcium signaling potency were detected between CXCL8(1-77), CXCL8(-2-77), CXCL8(2-77) and CXCL8(3-77) on cells transfected with one of the human CXCL8 receptors, i.e. CXCR1 and CXCR2. However, CXCL8(-2-77) was more potent compared to CXCL8(1-77), CXCL8(2-77) and CXCL8(3-77) in signaling and *in vitro* chemotaxis of peripheral blood-derived human neutrophils. Moreover, CXCL8(-2-77) was less efficiently processed by plasmin into the more potent CXCL8(6-77). The truncated forms CXCL8(2-77) and CXCL8(3-77) had higher affinity for heparin than CXCL8(1-77), a property important for the presentation of CXCL8 on endothelial layers. Upon intraperitoneal injection in mice, elongated, truncated and intact CXCL8 were equally potent to recruit neutrophils to the peritoneal cavity.

**Conclusions:**

In terms of their ability to induce neutrophil recruitment *in vivo*, the multiple CXCL8 forms may be divided in three groups. The first group includes CXCL8 proteins consisting of 75 to 79 amino acids, cleaved by aminopeptidases, with intermediate activity on neutrophils. The second group, generated through proteolytic cleavage (e.g. by Ser proteases), contains 69 to 72 amino acid forms which are highly potent neutrophil attractants *in vivo*. A third category is generated through the modification of the arginine in the NH_2_-terminal region into citrulline by peptidylarginine deiminases and has weak potency to induce neutrophil extravasation.

## Introduction

During infection, neutrophilic granulocytes are often the first immune cells to invade the inflammatory site. In addition to bacterial formyl peptides, complement fragment C5a and the lipid leukotriene B4, a number of chemokines with a conserved Glu-Leu-Arg (ELR) motif in front of the first cysteine (ELR^+^CXC chemokines) attract neutrophils [1], [2], [3]. All human ELR^+^CXC chemokines, i.e. CXCL1, CXCL2, CXCL3, CXCL5, CXCL6, CXCL7 and CXCL8 attract and activate neutrophils through the specific G protein-coupled CXC chemokine receptor CXCR2. In addition, CXCL6 and CXCL8 [or interleukin (IL)-8] also interact with CXCR1 [4]. Upon stimulation with bacterial or viral agents or inflammatory cytokines such as IL-1β or tumor-necrosis factor-α (TNF-α) CXCL8 can be produced by virtually any cell type including leukocytes, fibroblasts and epithelial and endothelial cells [5]. CXCL8 is one of the most abundant, if not the most abundant, ELR^+^CXC chemokines with the highest specific biological activity on neutrophils. CXCL8 may be transported across endothelial layers by the Duffy antigen receptor for chemokines (DARC) and presented to neutrophils in the blood flow on top of the glycocalyx through its interaction with glycosaminoglycans [1], [6].

To eradicate microbial infection, neutrophils use a number of innate immune mechanisms such as phagocytosis of bacteria and production of intracellular reactive oxygen components and hydrolyzing enzymes such as proteases that may be released in the extracellular milieu upon activation [7]. Recently, the formation of neutrophil extracellular traps (NETs), a mechanism that allows neutrophils to retain antimicrobial activity after death, has been identified as an additional mechanism and alternative for death by necrosis or apoptosis [8], [9]. A side effect of these efficient antimicrobial mechanisms and release of highly active molecules and hydrolyzing enzymes is partial tissue destruction. To provide the rapid and adequate immune response that is restricted in time to the duration of the infection and to avoid chronic inflammation, precise control of local neutrophil accumulation and activation is essential. One of the mechanisms that regulate neutrophil recruitment and activation is enzyme-induced posttranslational modification of ELR^+^CXC chemokines [10]. In fact, the ELR^+^CXC chemokine CXCL7 only becomes activated upon proteolytic removal of a large part of the NH_2_-terminal region [11], [12], [13]. For other ELR^+^CXC chemokines the activity has been reported to be up-regulated upon limited truncation of the NH_2_-terminus by specific enzymes such as plasmin, thrombin, matrix metalloproteases, etc. [10]. However, further truncation within the ELR motif results in almost complete inactivation of all ELR^+^CXC chemokines. Numerous posttranslationally modified natural forms of the different ELR^+^CXC chemokines have been identified and partially characterized [10]. Leukocyte-derived conditioned medium contains at least 10 different truncated and also citrullinated forms of the most potent human ELR^+^CXC chemokine, CXCL8 [14]. Incubation of CXCL8 with the myeloid aminopeptidase CD13 results in the removal of one or two amino acids from the 77 amino acid CXCL8(1-77) [15]. The Arg in position 5 is a crucial amino acid for cleavage of CXCL8 into CXCL8(6-77) by the serine proteases plasmin and thrombin. CXCL8 truncated by five to eight NH_2_-terminal residues, becomes a three- to ten-fold more potent neutrophil attractant and angiogenic molecule *in vitro* and *in vivo* [14], [16], [17]. Citrullination of natural CXCL8 by peptidylarginine deiminase (PAD)-2 or PAD-4 also occurs specifically on Arg in position 5 [14]. Citrullination significantly reduces the capacity of CXCL8 to induce neutrophil extravasation without affecting its angiogenic activity. However, intravenously injected citrullinated CXCL8 is a more potent mobilizer of mature neutrophils to the blood stream [18]. In addition to proteolytic truncation and citrullination, alternative cleavage of the signal peptide results in a natural CXCL8 form with two extra NH_2_-terminal amino acids, i.e. CXCL8 containing 79 amino acids or CXCL8(-2-77) [11], [14], [19], [20], [21].

Significant amounts of natural elongated CXCL8(-2-77) and truncated CXCL8(2-77) and CXCL8(3-77) have been reported to be produced by lymphocytes, monocytes and fibroblasts [11], [14], [19], [20], [21]. Since these forms co-elute on chromatographic columns with other CXCL8 forms the individual forms were not readily available as pure proteins for the evaluation of their biological activity. Here we produced the different CXCL8 forms by Fmoc solid phase peptide synthesis. The biological activity of three CXCL8 forms for which the activity was not investigated before, i.e. CXCL8(-2-77), CXCL8(2-77) and CXCL8(3-77), was compared with that of the previously studied CXCL8(1-77) and the more potent CXCL8(6-77) form in receptor binding, signaling and neutrophil migration assays.

## Results

### 1. Chemical synthesis of the naturally occurring CXCL8 variants

Separation of the natural CXCL8 variants CXCL8(-2-77), CXCL8(1-77), CXCL8(2-77), CXCL8(3-77) and CXCL8(6-77) via chromatography to study their biological activity was not feasible. These variants co-elute from analytical ion exchange and reversed-phase high performance liquid chromatography (RP-HPLC) columns [14], [20]. Therefore, the individual proteins were prepared by solid phase peptide synthesis, deprotected, folded and purified to homogeneity. The accuracy of the relative molecular mass (M_r_) of the synthesized proteins was verified by electrospray-ion trap mass spectrometry as shown in Table 1. Edman degradation-based NH_2_-terminal amino acid sequencing confirmed the NH_2_-terminal protein sequence of the synthesized chemokines (data not shown).

**Table 1:** Chemical synthesis of naturally occurring CXCL8 variants.

CXCL8 form^a^	NH_2_-terminal amino acid sequence	M_r_ (theoretical)	Experimentally determined M_r_ of synthetic CXCL8
CXCL8(-2-77)	_-2_EGAVLPRSAK ELR CQC	9104.7	9106.7
CXCL8(1-77)	_1_AVLPRSAK ELR CQC	8918.5	8918.8
CXCL8(2-77)	_2_VLPRSAK ELR CQC	8847.4	8847.6
CXCL8(3-77)	_3_LPRSAK ELR CQC	8748.3	8748.6
CXCL8(6-77)	_6_SAK ELR CQC	8381.8	8381.9

a CXCL8(-2-77), CXCL8(1-77), CXCL8(2-77), CXCL8(3-77) and CXCL8(6-77) were chemically synthesized as described in the Materials & Methods section. After purification, the M_r_ of the proteins was checked by electrospray ion trap mass spectrometry and the NH_2_-terminal amino acid sequence was confirmed by NH_2_-terminal sequencing based on Edman degradation.

### 2. Chemokine receptor-dependent activities of truncated CXCL8 variants

**Figure 1:**
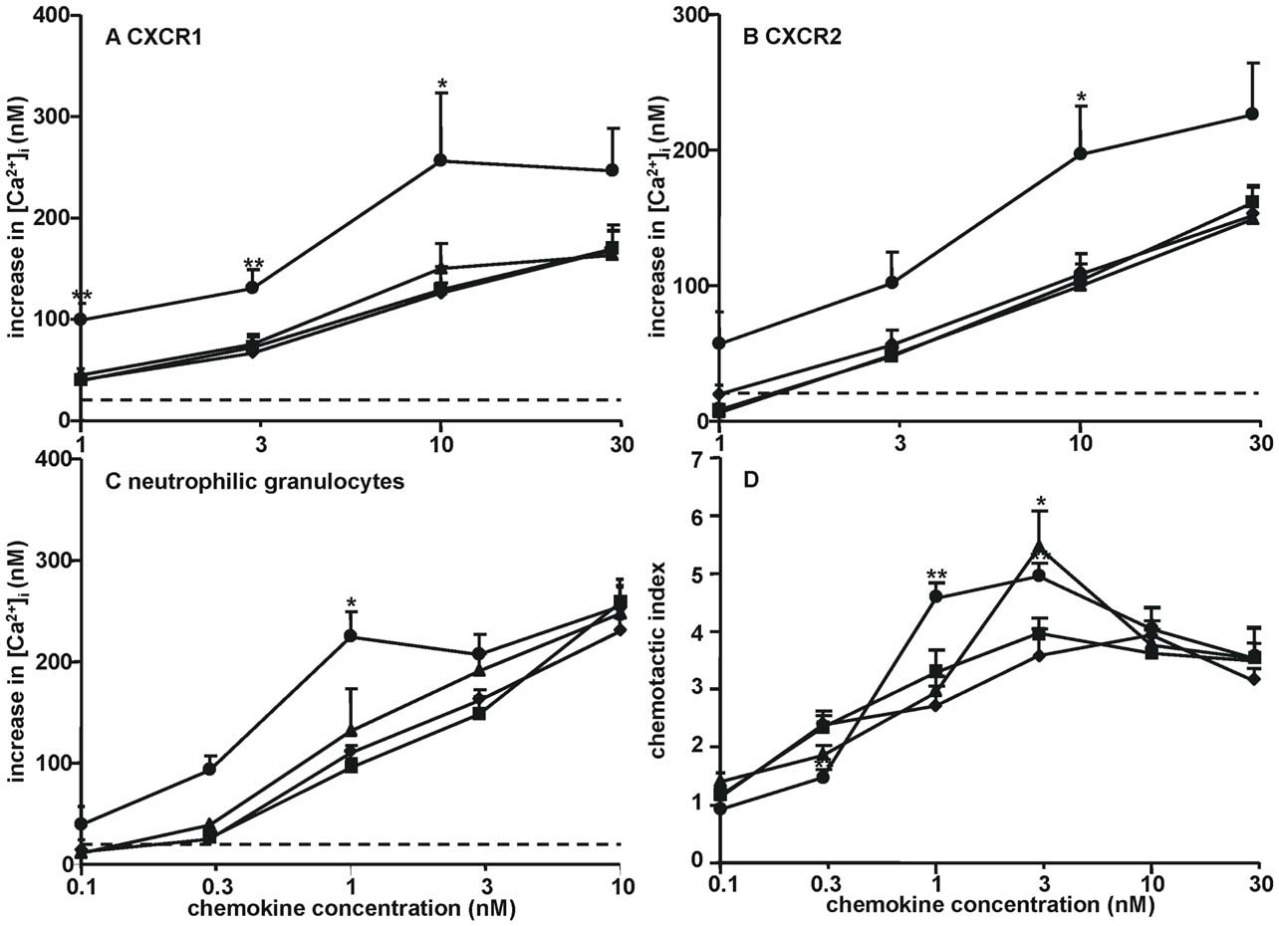
Chemokine receptor-dependent activity of the truncated CXCL8 isoforms *in vitro*. Intracellular calcium signaling upon activation of CXCR1 or CXCR2 was measured in HEK293 cells transfected with CXCR1 (panel A) or CXCR2 (panel B) or in neutrophilic granulocytes (panel C). Increases in the [Ca^2+^]_i_ (± SEM) are shown upon stimulation of the cells with the indicated concentrations of CXCL8(1-77) (♦), CXCL8(2-77) (▪), CXCL8(3-77) (▴) or CXCL8(6-77) (•). The dashed line indicates the detection limit (an increase in [Ca^2+^]_i_ of 20 nM). The Mann-Whitney U test was used to detect statistical differences [*, p<0.05; **, p<0.01; compared to the corresponding concentration of CXCL8(1-77)] (n≥3). The neutrophil chemotactic activity of CXCL8(1-77) (♦), CXCL8(2-77) (▪), CXCL8(3-77) (▴) and CXCL8(6-77) (•) was evaluated in 96-well Boyden chambers (panel D). Values represent the mean chemotactic index (± SEM). Statistical analysis was performed using the Mann-Whitney U test [*, p<0.05; **, p<0.01; compared to the corresponding concentration of CXCL8(1-77)] (n = 6).

CXCL8 is a potent neutrophil-activating and -attracting chemokine, binding to CXCR1 and CXCR2. Following activation of one of these receptors, a rapid increase in the intracellular calcium concentration ([Ca^2+^]_i_) is elicited, a process crucial for the induction of several cellular responses (related and unrelated to neutrophil migration). The calcium signaling potencies of the truncated CXCL8 variants were compared with intact CXCL8(1-77) (Figure 1A, 1B, 1C). The effects of truncation on the signaling activity of CXCL8 were similar on CXCR1-transfected HEK293 cells, CXCR2-transfected HEK293 cells and freshly isolated human blood neutrophils. As expected, CXCL8(6-77) was a three- to ten-fold more potent agonist of CXCR1 and CXCR2 than CXCL8(1-77). In contrast, more limited NH_2_-terminal truncation, generating CXCL8(2-77) or CXCL8(3-77), did not influence the signaling potency of CXCL8. The dose-response curves of CXCL8(1-77), CXCL8(2-77) and CXCL8(3-77) coincided in CXCR1-transfected cells, CXCR2-transfected cells and neutrophils, the minimal effective concentration inducing an increase in [Ca^2+^]_i_ being 1 nM and 3 nM on CXCR1- and CXCR2-transfected HEK293 cells, respectively. Calcium responses in fresh human neutrophils were already detectable upon stimulation with 0.3 nM CXCL8(1-77), CXCL8(2-77) and CXCL8(3-77).

Analogous results were obtained when comparing the *in vitro* neutrophil chemotactic activity of these CXCL8 variants (Figure 1D), yielding similar bell-shaped dose-response curves for CXCL8(1-77), CXCL8(2-77) and CXCL8(3-77). The efficacy (the maximal chemotactic response) of CXCL8(6-77) was higher than that of CXCL8(1-77) (chemotactic index 4.75 vs. 3.76) and was reached at three-fold lower CXCL8 concentrations (1 vs. 3 nM). Remarkably, CXCL8(3-77) was more efficacious compared to CXCL8(1-77). It reached a significantly higher chemotactic index at the concentration resulting in the optimal chemotactic response (3 nM).

### 3. Chemokine receptor-dependent activity of the elongated CXCL8 variant

**Figure 2:**
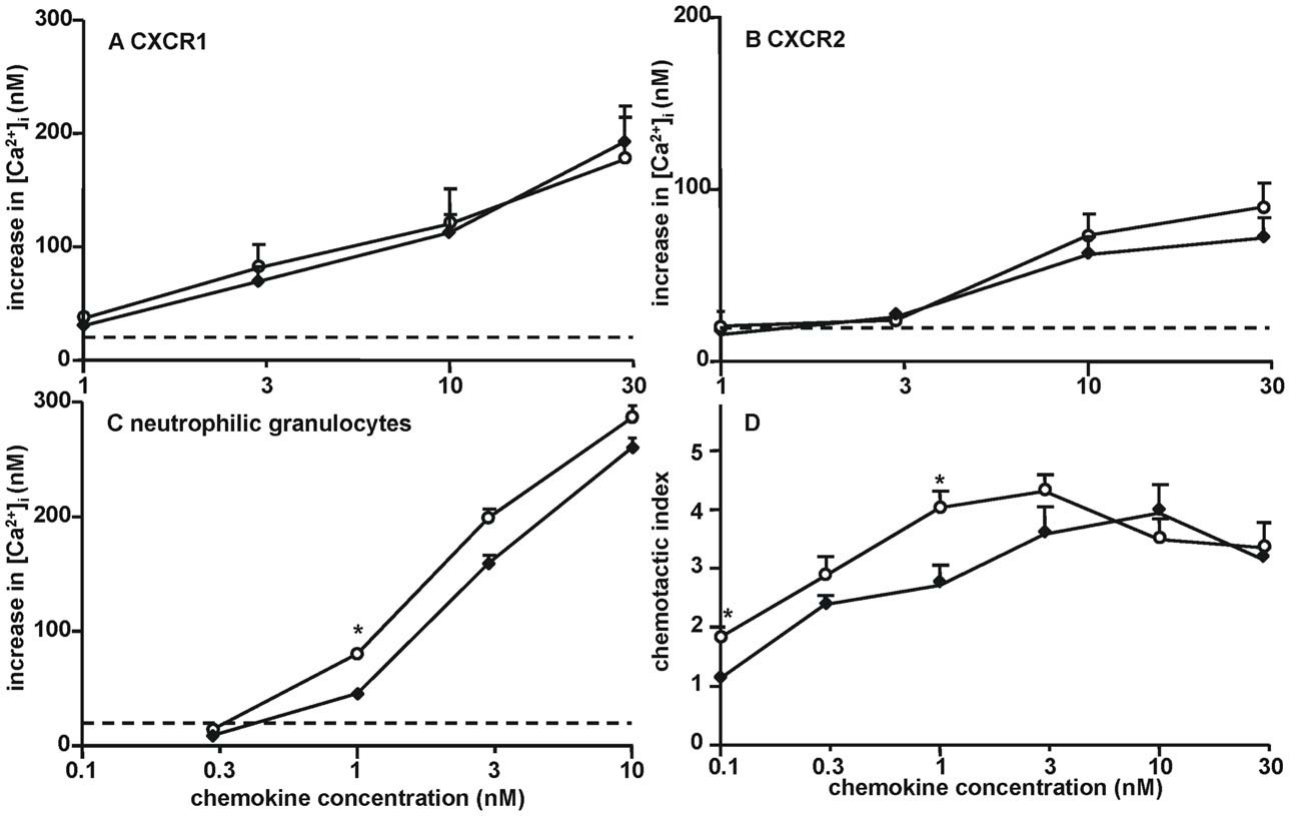
Chemokine receptor-dependent activity of the elongated CXCL8 variant *in vitro*. Intracellular calcium signaling upon activation of CXCR1 or CXCR2 was measured in HEK293 cells transfected with CXCR1 (panel A) or CXCR2 (panel B) or in neutrophilic granulocytes (panel C). Increases of the [Ca^2+^]_i_ (± SEM) are shown upon stimulation of the cells with the indicated concentrations of CXCL8(1-77) (♦) or CXCL8(-2-77) (○). The dashed line indicates the detection limit (an increase in [Ca^2+^]_i_ of 20 nM). The Mann-Whitney U test was used to detect statistical differences [*, p<0.05; compared to the corresponding concentration of CXCL8(1-77)] (n≥3). The neutrophil chemotactic activity of CXCL8(1-77) (♦) or CXCL8(-2-77) (○) was evaluated in 96-well Boyden chambers (panel D). Values represent the mean chemotactic index (± SEM). Statistical analysis was performed using the Mann-Whitney U test [*, p<0.05; compared to the corresponding concentration of CXCL8(1-77)] (n = 6).

The calcium signaling potency of CXCL8(1-77) was also compared with that of the elongated isoform of CXCL8, i.e. CXCL8(-2-77). As shown in Figure 2 (panel A, B and C), signaling through CXCR1 or CXCR2 was comparable for CXCL8(1-77) and CXCL8(-2-77). However, on neutrophils CXCL8(-2-77) appeared to be a slightly more potent inducer of calcium mobilization. Indeed, 1 nM CXCL8(-2-77) induced a significantly higher increase in [Ca^2+^]_i_ compared to 1 nM CXCL8(1-77). In addition, for CXCL8(-2-77) a three-fold lower concentration was required *in vitro* to induce a comparable response in neutrophil chemotaxis assays compared to CXCL8(1-77) (Figure 2D).

### 4. Binding properties of the CXCL8 variants

**Figure 3:**
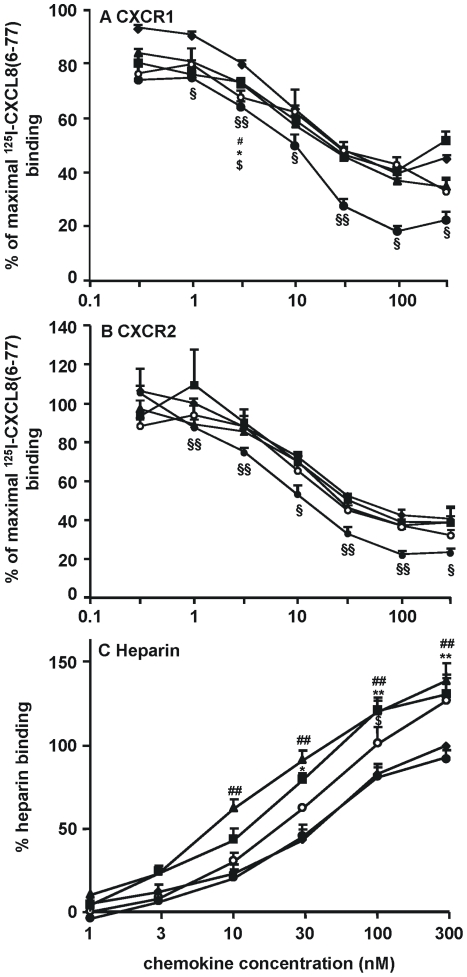
Binding properties of the CXCL8 isoforms. HEK293 cells transfected with CXCR1 (A) or CXCR2 (B) were incubated with increasing concentrations of unlabeled CXCL8(-2-77) (○), CXCL8(1-77) (♦), CXCL8(2-77) (▪), CXCL8(3-77) (▴) or CXCL8(6-77) (•), together with ^125^I-labeled CXCL8(6-77). The mean remaining % of ^125^I-CXCL8(6-77) binding (± SEM) is specified at the y-axis [n≥4 (1 to 300 nM); n = 2 (0,3 nM)]. Analysis of the interaction of the CXCL8 variants with heparin was performed using heparin binding plates. The indicated concentrations of CXCL8(-2-77) (○), CXCL8(1-77) (♦), CXCL8(2-77) (▪), CXCL8(3-77) (▴) and CXCL8(6-77) (•) were added to immobilized heparin, the binding equilibrium was achieved and bound CXCL8 was detected with labeled antibodies. The mean (from four to six independent experiments) percentage heparin binding [compared to 300 nM CXCL8(1-77)] (± SEM) for the individual CXCL8 forms is indicated at the y-axis. To detect statistically significant differences between the CXCL8 variants and CXCL8(1-77), the Mann-Whitney U test was carried out [$,*,#,§, p<0.05; **,##,§§, p<0.01; for comparison of CXCL8(-2-77) ($), CXCL8(2-77) (*), CXCL8(3-77) (#) and CXCL8(6-77) (§) with CXCL8(1-77)].

To evaluate the binding efficiency of the different CXCL8 isoforms to the G protein-coupled receptors CXCR1 and CXCR2, their ability to compete for ^125^I-labeled CXCL8(6-77) binding to these receptors was compared (Figure 3A and 3B). CXCL8(1-77) competed with radiolabeled CXCL8 in a dose-dependent manner for binding to both CXCR1 and CXCR2. The NH_2_-terminal variants CXCL8(2-77), CXCL8(3-77) and CXCL8(-2-77) were as potent as CXCL8(1-77) in displacing radiolabeled CXCL8 from either CXCR1 or CXCR2. In contrast to these isoforms, but confirming previously published results [14], CXCL8(6-77) displayed increased binding affinity for both CXCR1 and CXCR2 as it displaced radiolabeled CXCL8 about 3-fold more efficiently. Remarkably, on CXCR1-transfected cells and at low concentrations, CXCL8(2-77), CXCL(3-77) and CXCL8(-2-77) seem to be more potent competitors compared to CXCL8(1-77). However, this difference is significant at 3 nM only.

Glycosaminoglycan binding is indispensable for chemokines in order to build up a chemotactic gradient *in vivo* and to be presented on the endothelial glycocalyx. The heparin binding affinity of the CXCL8 variants was compared in an enzyme-linked immunosorbent saturation binding assay (Figure 3). The previously reported equal heparin binding affinity of CXCL8(1-77) and CXCL8(6-77) was confirmed [14]. Furthermore, this assay showed that the truncated CXCL8 variants CXCL8(2-77) and CXCL8(3-77) displayed about three-fold higher binding affinities for heparin than CXCL8(1-77). Despite the presence of an extra negatively charged acidic NH_2_-terminal amino acid (i.e. Glu), the heparin binding efficiency of CXCL8(-2-77) was moderately higher than the binding efficiency of CXCL8(1-77).

### 5. Susceptibility of CXCL8 forms to cleavage by thrombin and plasmin

**Figure 4:**
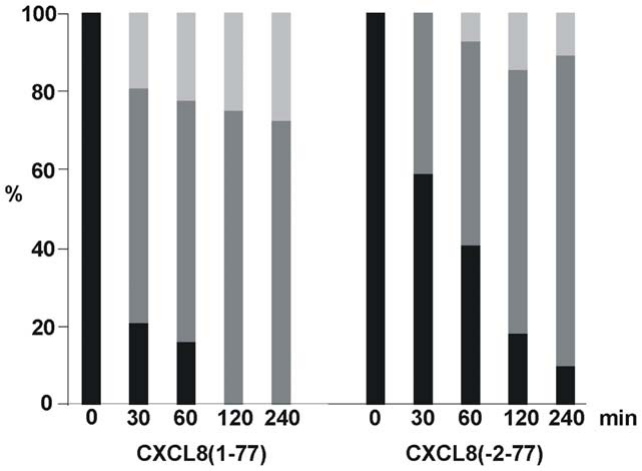
Susceptibility of CXCL8(-2-77) to proteolysis by plasmin. CXCL8(1-77) and CXCL8(-2-77) were incubated with plasmin and the degree of conversion was determined using electrospray ion trap mass spectrometry. Percentages of intact CXCL8 [either CXCL8(1-77) or CXCL8(-2-77)] (black histograms), CXCL8(6-77) (dark grey histograms) and CXCL8(9-77) (light grey histograms) generated upon incubation with plasmin are depicted in function of time (min).

The plasma serine proteases plasmin and thrombin cleave CXCL8(1-77) in a very efficient way, thereby potentiating its neutrophil chemotactic activity [22], [23]. Plasmin has a preference for the peptide bond between Arg^5^ and Ser^6^, although minor cleavage of the Lys^8^-Glu^9^ bond also occurs, whereas thrombin specifically cleaves the Arg^5^-Ser^6^ bond of CXCL8. To study whether NH_2_-terminal truncation by one or two amino acids or the additional Glu-Gly dipeptide hinder or promote plasmin- or thrombin-mediated cleavage, processing of CXCL8(1-77), CXCL8(2-77), CXCL8(3-77) and CXCL8(-2-77) by these proteases was analyzed by mass spectrometry. All four substrates were converted with comparable efficiency by thrombin (data not shown), showing that limited variation of the length of the CXCL8 NH_2_-terminus did not affect the cleavage rate of thrombin. Similarly, the susceptibility of CXCL8 for cleavage by plasmin was not changed by limited NH_2_-terminal truncation. In contrast, plasmin cleaved CXCL8(-2-77) less efficiently compared to CXCL8(1-77) (Figure 4). Under the experimental conditions described in the Materials & Methods section, approximately 41% of CXCL8(-2-77) versus 79% of CXCL8(1-77) had undergone proteolysis by plasmin after 30 min of incubation. Complete conversion of CXCL8(1-77) was achieved upon 60 min of incubation, whereas at that moment more than 40% of CXCL8(-2-77) was still intact. This difference in susceptibility may influence the biological activity of CXCL8(-2-77) *in vivo*.

### 6. Neutrophil migration *in vivo* induced by CXCL8 forms

**Figure 5:**
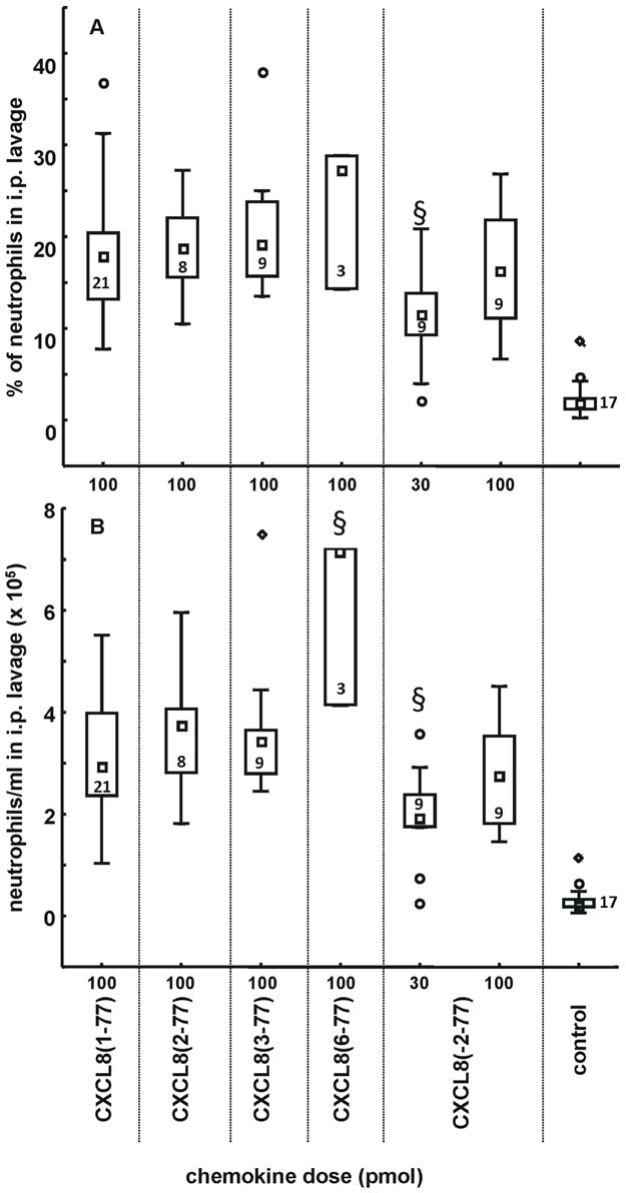
Neutrophil-attracting activity of the CXCL8 isoforms *in vivo*. The neutrophil influx upon i.p. injection of the specified doses of the CXCL8 isoforms in mice was investigated. 2 hours post-injection, the peritoneal cavity was washed and the overall number of leukocytes determined. Differential microscopic counting of Hemacolor-stained cells allowed to define the percentage of neutrophils (panel A) and the total number of neutrophils (panel B) present in the peritoneal cavity. Squares show the median neutrophil influx (formulated as percentages or total counts/ml); the bottom and the top of the rectangle denote the 25th and 75th percentile; whiskers represent the non-outlier range (coefficient 1.5). Outliers are plotted as circles. Control mice (co) were treated with vehicle and illustrate the spontaneous migration of neutrophils to the peritoneal cavity. The Mann-Whitney U test was used for statistical analysis [§, p<0.01; compared with 100 pmol CXCL8(1-77)]. The numbers depicted in (or besides) the rectangular box represent the number of mice treated.

The *in vivo* neutrophil recruitment potency of the NH_2_-terminal CXCL8 variants was compared with that of CXCL8(1-77). Although no homologous murine CXCL8 equivalent exists, human CXCL8 activates murine neutrophils. Mice were intraperitoneally (i.p.) injected with the CXCL8 forms and after 2 h the peritoneal cavity was washed and the number of leukocytes and the percentage of neutrophils were determined. In this *in vivo* model, the neutrophil influx induced by CXCL8(1-77), CXCL8(2-77), CXCL8(3-77) and CXCL8(-2-77) was comparable (Figure 5). Indeed, injection (i.p.) of 100 pmol of either of these isoforms elevated the number of neutrophils in the peritoneal cavity from about 2.10^4^ neutrophils/ml (upon vehicle injection) to approximately 30.10^4^ neutrophils/ml. As a positive control, CXCL8(6-77) was included in this assay. The higher potency of CXCL8(6-77) compared to CXCL8(1-77) could be confirmed, as the median number of neutrophils present in the intraperitoneal lavage upon injection of 100 pmol of CXCL8(6-77) is significantly higher than upon injection of 100 pmol of CXCL8(1-77) (70.10^4^ neutrophils/ml vs. 30.10^4^ neutrophils/ml). Thus, no difference in neutrophil recruitment potency was detected between CXCL8(1-77), CXCL8(2-77), CXCL8(3-77) and CXCL8(-2-77) in this *in vivo* assay in mice, despite the small, but significant differences observed in calcium mobilization, chemotaxis and heparin binding assays and regarding the sensitivity to proteolytic activation by plasmin (*vide supra*).

## Discussion

IL-8 or CXCL8 is the most potent human chemokine with neutrophil chemotactic properties. Already upon the initial isolation of natural human CXCL8 it was clear that several different NH_2_-terminal isoforms of CXCL8 are produced by leukocytes or fibroblasts under inflammatory conditions [11], [19], [20], [21], [22]. Posttranslational modifications can alter the biological activity (receptor affinity and signaling potency), interaction with other molecules (e.g. glycosaminoglycans), clearance of chemokines and hence the *in vivo* inflammatory properties [10]. The most abundant CXCL8 isoforms CXCL8(1-77) and CXCL8(6-77), produced by endothelial cells [24], [25], fibroblasts [19] and monocytes [21], [26] respectively, have been studied extensively. These studies revealed that the removal of five NH_2_-terminal amino acids (AVLPR) potentiates the neutrophil chemotactic activity of CXCL8 both *in vitro* and *in vivo* [14], [22], [23]. Additional limited NH_2_-terminal truncations to CXCL8(8-77) and CXCL8(9-77) gradually increase the chemotactic potency *in vitro*, suggesting that CXCL8(6-77) is not the most active form of CXCL8 [16], [27]. A crucial role in receptor binding and activation has been ascribed to the ELR motif in front of the CXC sequence using both chemically synthesized truncated and mutated analogs [16], [28]. Thus far, truncation of the NH_2_-terminus of CXCL8 has been shown to result in potentiation as long as proteases do not cleave in and beyond the ELR motif.

In this study, the elongated natural variant of CXCL8, i.e. CXCL8(-2-77) and the truncated forms CXCL8(2-77) and CXCL8(3-77) were characterized. The elongated form is presumed to arise from alternative cleavage of the signal peptide of the 99 amino acid precursor [29]. It has been detected in medium of peripheral blood mononuclear cells (PBMCs) conditioned with lipopolysaccharide (LPS), concanavalin A or a combination of polyriboinosinic polyribocytidylic acid (PIC) and interferon-γ, where it constitutes about 8 to 10% of the total amount of CXCL8 produced [11], [14], [20], [21]. In addition, the supernatant of cultured IL-1α- or TNF-α-stimulated dermal fibroblasts contains significant amounts of CXCL8(-2-77) [19]. Albeit less abundant, the truncated forms CXCL8(2-77) and CXCL8(3-77) have also been isolated from the conditioned medium of PBMCs [14]. These forms may result from aminopeptidase (e.g. CD13)-mediated cleavage of secreted CXCL8(1-77) [15].

The results presented here show that the NH_2_-terminally different isoforms described do not differ a lot in their effects on binding to and signaling through CXCR1 or CXCR2. The receptor binding and calcium signaling potency of CXCL8(1-77), CXCL8(2-77), CXCL8(3-77) and CXCL8(-2-77) is similar in both CXCR1- or CXCR2-transfected cells. However, in calcium signaling and *in vitro* chemotaxis assays on freshly isolated blood neutrophils, expressing both CXCR1 and CXCR2, stronger responses to stimulation with CXCL8(-2-77) than towards stimulation with CXCL8(1-77) were observed. Moreover, small alterations in the NH_2_-terminal region do influence the GAG binding affinity of CXCL8. CXCL8(2-77) and CXCL8(3-77) displayed a three-fold higher affinity for heparin compared to CXCL8(1-77). Although not as explicit as the truncated isoforms, CXCL8(-2-77) also bound heparin with higher affinity than CXCL8(1-77). Conversion of CXCL8 into the more potent neutrophil chemoattractant CXCL8(6-77) by the serine proteases plasmin and thrombin was not affected by prior removal of the NH_2_-terminal amino acids Ala^1^ or Val^2^. This is in contrast to CXCL8 citrullination which prevented subsequent CXCL8 activation by these serine proteases [14]. On the other hand, CXCL8(-2-77), generated through alternative cleavage of the signal peptide, was less susceptible to cleavage by plasmin. Finally, the neutrophil-attracting activity of CXCL8(-2-77), CXCL8(1-77), CXCL8(2-77) and CXCL8(3-77) were evaluated *in vivo* upon i.p. injection in mice. During *in vivo* leukocyte migration, many more parameters come into play, including glycosaminoglycan binding and alteration of the expression pattern of adhesion molecules such as selectins and integrins. Despite the slightly increased *in vitro* chemotactic potency and GAG binding affinity, the *in vivo* neutrophil-attracting potency of CXCL8(-2-77) upon i.p. injection in mice did not differ from that of CXCL8(1-77). Injection of 100 pmol of CXCL8(1-77) or 100 pmol of CXCL8(-2-77) both elevated the percentage of neutrophils in the peritoneal cavity from about 2% to 18%. Perhaps, the higher variability of *in vivo* assays prohibits detection of small differences in biological activity. Alternatively, the reduced susceptibility of CXCL8(-2-77) to cleavage and activation by plasmin may counteract the increased chemotactic potency and GAG binding property of CXCL8(-2-77). Although alternative cleavage of the signal peptide of the CXCL8 precursor was reported in various publications, it does not seem to have an effect on the *in vivo* neutrophil-attracting activity of CXCL8 and thus, does not constitute a major regulatory mechanism. Furthermore, although NH_2_-terminal removal of amino acids one by one from CXCL8(6-77) was reported to improve the activity *in vitro* [16], aminopeptidase treatment of CXCL8(1-77) resulting in the generation of CXCL8(2-77) and CXCL8(3-77) did not progressively generate more potent isoforms *in vivo* compared to CXCL8(1-77).

**Figure 6:**
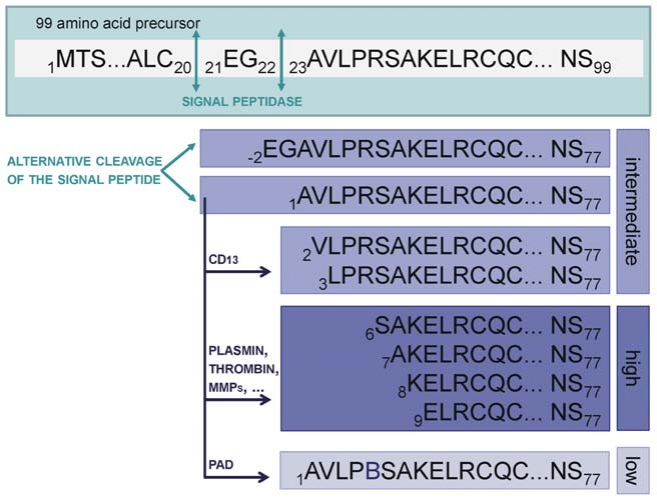
Overview of the naturally occurring CXCL8 isoforms. Several NH_2_-terminally modified isoforms of CXCL8 have been purified from the conditioned medium of leukocytes, fibroblasts and endothelial cells. The NH_2_-terminal and COOH-terminal sequences of these isoforms are depicted in the one-letter code (B = citrulline). Based on the criteria formulated by Von Heijne [29], the 99 amino acid precursor molecule may be cleaved by signal peptidases between Cys_20_ and Glu_21_ or between Gly_22_ and Ala_23_, the latter being more probable. Indeed both resulting CXCL8 proteins have been identified and are indicated in this manuscript as CXCL8(-2-77) and CXCL8(1-77), respectively. CXCL8(1-77) can be cleaved by a number of proteases, resulting in the listed NH_2_-terminally truncated forms. Furthermore, CXCL8 can be citrullinated by peptidylarginine deiminase (PAD). These NH_2_-terminal modifications differently affect the ability of CXCL8 to recruit neutrophils. The *in vivo* neutrophil-attracting activity is defined in the boxes at the right. CXCL8(-2-77), CXCL8(1-77), CXCL8(2-77) and CXCL8(3-77) show comparable intermediate activity. The more extensively truncated isoforms CXCL8(6-77), CXCL8(7-77), CXCL(8-77) and CXCL8(9-77), in contrast, display enhanced neutrophil recruiting potency, whereas citrullination significantly lowers the capacity of CXCL8 to guide neutrophils. (MMP, matrix metalloprotease).

Combining these results with the results previously described for CXCL8(6-77) and [Cit^5^]CXCL8(1-77) [14], we can conclude that, based on their potency to recruit neutrophils, the NH_2_-terminal variants of CXCL8 can be divided into 3 subgroups (Figure 6). A first group contains CXCL8(-2-77), CXCL8(1-77), CXCL8(2-77) and CXCL8(3-77), which display intermediate neutrophil-attracting capacity. Upon removal of 5 to 8 NH_2_-terminal amino acids, CXCL8 more efficiently attracts neutrophils, therefore these isoforms [i.e. CXCL8(6-77), CXCL8(7-77), CXCL8(8-77) and CXCL8(9-77)] form a second group with enhanced biological activity. Citrullinated CXCL8 belongs to a third category as it displays no or low neutrophil-attracting potency. The significant differences in terms of their ability to recruit neutrophils, point towards the importance of differential detection of these individual isoforms in patient samples and towards a potentially important role for CXCL8-modifying enzymes such as plasmin, thrombin and PADs in fine-tuning neutrophil migration under pathological conditions. Quantification of the individual CXCL8 forms in patient samples will help to unveil the potential pathophysiological role of these different CXCL8 isoforms.

## Materials and Methods

### Ethics statement

The experimental protocols (involving the use of laboratory animals) were carried out in strict accordance with the Belgian and European guidelines concerning the handling of laboratory animals and were approved by the Animal Ethical Committee of the K.U.Leuven (approved project P07029).

No specific approval from an institutional review board is required for the use of buffy coats for the following reasons: (1) no personal patient information is made available, (2) buffy coats cannot be used for treatment of patients and are waste products for the blood transfusion centre and (3) blood donors sign an agreement that parts of the donation that cannot be used for patient treatment may be used for scientific research.

### Reagents and cells

Recombinant human CXCL8(1-77) and CXCL8(6-77) were obtained from PeproTech (Rocky Hill, NJ, USA). Thrombin and plasmin purified from human plasma were purchased from Sigma-Aldrich (St. Louis, MO, USA). Human embryonic kidney (HEK) 293 cells transfected with human CXCR1 or CXCR2 were provided by Dr. J. M. Wang (National Cancer Institute, Frederick, MD) [30] and cultured in Dulbecco's modified Eagle's medium (DMEM; Lonza, Basel, Switzerland) supplemented with 10% (v/v) fetal bovine serum (FBS) and 800 µg/ml geneticin (Invitrogen, Merelbeke, Belgium).

### Enzymatic modification of CXCL8 in vitro

To evaluate the susceptibility of CXCL8 variants to enzymatic modification by plasmin or thrombin, human CXCL8 variants were incubated with thrombin or plasmin at 37°C for the indicated time intervals at enzyme-substrate (E/S) molar ratios of 1/100 and 1/1000, respectively. Cleavage was accomplished in phosphate-buffered saline (PBS) (pH 7.4). CXCL8 processing was stopped by adding 0.1% (v/v) trifluoroacetic acid (TFA). Processed preparations were desalted on C4 ZipTips (Millipore, Billerica, MA, USA) and analyzed by electrospray ion trap mass spectrometry (Esquire LC ion trap mass spectrometer; Bruker, Bremen, Germany).

### Chemical synthesis of CXCL8 and the posttranslationally modified isoforms

CXCL8(-2-77), CXCL8(1-77), CXCL8(2-77), CXCL5(3-77) and CXCL8(6-77) were chemically synthesized based on fluorenyl methoxycarbonyl (Fmoc) chemistry using a 433A solid phase peptide synthesizer (Applied Biosystems, Foster City, CA, USA), as described by Loos et al. [31]. Intact synthetic peptides were separated from incompletely synthesized by-products by RP-HPLC on a Source 5-RPC column (4.6×150 mm; GE Healthcare Bio-sciences, Little Chalfont, United Kingdom) applying an acetonitrile gradient in 0.1% (v/v) TFA. Part (0.7%) of the column effluent was split online to an ion trap mass spectrometer (Esquire LC). Fractions containing the protein with the correct M_r_ were folded overnight, subsequently loaded on a C8 RP-HPLC column (Aquapore-RP 300 2.1×220 mm; PerkinElmer, Waltham, MA, USA) and eluted with an acetonitrile gradient in 0.1% TFA. Proteins eluting from the column were detected spectrophotometrically at 214 nm and the M_r_ was measured online by ion trap mass spectrometry. Homogenous CXCL8 fractions were pooled, lyophilized and dissolved in ultrapure water (MilliQ; Millipore). Several assays were applied to determine protein concentrations, i.e. enzyme-linked immunosorbent assay (ELISA), NH_2_-terminal sequencing based on Edman degradation (491 cLC protein sequencer, Applied Biosystems), sodium dodecyl sulfate-polyacrylamide gel electrophoresis (SDS-PAGE) and bicinchoninic acid (BCA) protein assay (Pierce, Rockford, IL, USA).

### Isolation of granulocytes from fresh human buffy coats

Fresh human buffy coats were obtained from the Blood Transfusion Center of the Red Cross (Leuven, Belgium). The bulk of erythrocytes were removed by sedimentation in erythrocyte-aggregating hydroxyethyl starch (Plasmasteril, Fresenius AG, Bad Homburg, Germany) for 30 min. After centrifugation, the leukocyte pellet was dissolved in PBS and layered on Ficoll-sodium diatrizoate medium (Lymphoprep, Axis-Shield PoC AS, Oslo, Norway) in order to separate mononuclear cells from granulocytes by density gradient centrifugation (30 min, 400×g). Residual erythrocytes in the granulocyte pellet were eliminated by hypotonic shock (30 s) in ultrapure water (MilliQ). Purified granulocytes were used for chemotaxis and calcium signaling experiments.

### Calcium mobilization through CXCR1 and CXCR2 by CXCL8

The capacity of the CXCL8 isoforms to induce signaling through human CXCR1 or human CXCR2 resulting in an increase of the [Ca^2+^]_i_ was tested on both freshly purified granulocytes and CXCR1- and CXCR2-transfected HEK293 cells, as described [32].

### Neutrophil migration in vitro and in vivo in response to CXCL8

The chemotactic activity of the different CXCL8 isoforms for neutrophilic granulocytes *in vitro* was compared using a 96-well Boyden chamber-based chemotaxis assay (Multiscreen plates, Millipore) and migrated neutrophils were quantified with a luminescence ATP detection assay (PerkinElmer), as previously described [32].

*In vivo* blood vessel extravasation of circulating neutrophilic granulocytes into the peritoneal cavity was examined by i.p. injection of endotoxin-free CXCL8 [in 0.9% (w/v) NaCl] or vehicle in female NMRI mice (8–10 weeks, raised pathogen-free; Elevage Janvier, Le Genest Saint Isle, France). Endotoxin concentrations were evaluated with the *Limulus* amoebocyte lysate test (Cambrex, East Rutherford, NJ, USA). After 2 h, mice were sacrificed and the peritoneal cavity was washed with 5 ml saline enriched with 2% (v/v) FBS and 20 U/ml heparin. Total leukocyte counts in the peritoneal lavage were determined. 10^5^ cells were used for the preparation of two cytospins/mouse. After Hemacolor (Merck, Darmstadt, Germany) staining, percentages of neutrophils in the peritoneal lavage were determined in duplicate by differential microscopic counting of at least 100 leukocytes per cytospin.

### Binding of CXCL8 to chemokine receptors CXCR1 and CXCR2

The binding capacity of the CXCL8 isoforms was evaluated through the measurement of competition with ^125^I-labeled CXCL8(6-77) (PerkinElmer). CXCR1- or CXCR2-transfected cells (2×10^6^), reconstituted in binding buffer (50 mM HEPES, 1 mM CaCl_2_, 5 mM MgCl_2_ and 0.1% (w/v) bovine serum albumin), were incubated for 2 h with radiolabeled CXCL8 and varying concentrations of unlabeled CXCL8. Subsequently, the cells were washed twice with 1 ml of binding buffer. Finally, gamma radiation emitted by the cells was measured with a gamma counter. Maximal ^125^I-CXCL8(6-77) binding was determined by incubating the cells with radiolabeled CXCL8 alone and was set at 100%.

### Binding of CXCL8 to heparin

The heparin binding properties of the CXCL8 isoforms were compared on heparin binding plates (Iduron, Paterson Institute for Cancer Research, University of Manchester, Manchester, UK), which adsorb heparin without modification to retain the protein-binding characteristics. Heparin (Low in calcium; Iduron), diluted in standard assay buffer (SAB; 100 mM NaCl, 50 mM sodium acetate, 0.2% v/v Tween-20; pH 7.2) (25 µg/ml) was coated overnight at room temperature on the plasma-polymerized surface of heparin binding plates. After three wash steps with SAB, the plates were blocked with blocking buffer (SAB enriched with 0.2% w/v gelatin) for 1 h at 37°C. Blocking buffer was discarded and dilutions of CXCL8 (in blocking buffer) were added in triplicate and incubated for 2 h at 37°C. Unbound CXCL8 was removed by three washes with SAB and bound CXCL8 was detected with biotinylated polyclonal rabbit anti-human CXCL8 (PeproTech), diluted in blocking buffer. After 1 h of incubation at 37°C, the plates were washed with SAB. Horse radish peroxidase-labeled streptavidin was added for 30 min at 37°C. Subsequently, the plates were washed and the peroxidase activity was quantified by adding a horse radish peroxidase substrate solution and conversion of 3,3′,5,5′-tetramethylbenzidine, supplemented with 0.004% (v/v) H_2_O_2_ was measured at 450 nm using a spectrophotometer. Equal affinity of the biotinylated anti-human CXCL8 antibody for the different CXCL8 variants was verified. Percentage heparin binding was calculated by subtracting the mean optical density (OD) of the control samples (blocking buffer) from the average OD of the test sample, followed by division by the average OD obtained with 300 nM of CXCL8(1-77) and multiplication by 100.
